# Supplementary material for: Disparities in childhood composite index of anthropometric failure prevalence and determinants across Ethiopian administrative zones
Source: PLoS One. 2021 Sep 23;16(9):e0256726. doi: 10.1371/journal.pone.0256726 (PMC8459952; doi:10.1371/journal.pone.0256726)
Supplement: Attachment — Submitted filename: Comments.docx [file pone.0256726.s001.docx]

Overall, the manuscript is interesting. However, the manuscript might benefit from some revision.

The authors need to show a clear constructing of the composite measure index and compare the composite measure index and the three traditional measures indices (WAZ, HAZ, WHZ). Also, authors should address the question, "what does the difference between the composite indicator measure and the traditional once? In addition, as they using hierarchical data structure and generalized multilevel mixed model is selected (line 182), the authors need to provide a clear level of the model and the notation of the variables in each level in the method section.

DHS **children's data contains** children of women born in the last 5 years, hence, authors how the correlated effects of children from the same women treated? It needs clear explanations. On top of these, the title is “Disparities in childhood Composite Index of Anthropometric Failure prevalence determinants across Ethiopian administrative Zones over time” but the effect of time is not investigated/showed and hence authors should indicated the effect of time on the CIAF prevalence in their results.

Minor: all figures and tables are not clear and needs re-organize with PLOS ONE format. And the references are not following the journal format (e.g.: it is found in font size of 11 and single spacing). Abbreviations with its long form should state at the first time and used only abbreviations after a while, for instance “Composite Index of Anthropometric Failure (CIAF)” is used in whole document with similar fashion whereas “U5C” is in other round way.

Abstract:

1. Line 6-8: “…..in 2000, 2005, 2011, and 2016” should read “…. (EDHSs) of 2000, 2005, 2011 and 2016, a population-based cross-sectional study of 29,599 under-five year children from 63 Zones.”
2. Line 14: “The pooled prevalence of CIAF…..” what “Pooled” indicates here? CIAF is already indicates the aggregate value, thus authors should read as “the prevalence of CIAF….”

Introduction:

1. Line 39-41: “….different forms of malnutrition” should read “….different forms of undernutrition”. Because, CIAF is computed from wasting, stunting and underweight only and malnutrition is beyond these three measures.
2. Line 55-57: “As the administrative Zones are mainly ethnic-based, ….” Is unrelated and unclear statement and should remove from the section.

Methods:

1. Line 74-75: “This study was conducted on 29,599 children consisting……” does the study unit children?
2. Authors indicate somewhere predictors are considered from child level and women level and the linear complement of the model is given at line 110 wrongly. This is because, child level and women level predictors are not included in the model clearly. Hence it needs clarification!
3. Line 119-128: all of these lines are reading about BLUP, the model that authors used in this manuscript is GLMM and the parameter of such model is estimated by either MML or IRWLS or other but not by BLUP. Thus, authors are used wrong methods for parameter estimation and needs critical correction. In general, the model and parameter estimation method is not appropriate for the data and needs refitting and re-estimating the parameters using appropriate model (GMMM) and estimation method (MML or IGWLS or nay other).

Result:

1. It would have been useful for the authors to show what percentage or proportion children were classified as undernourished using each of the measures separately and what new information the composite measure is contributing.
2. Line 132: “ …. in the years between 2000 and 2016 in Ethiopia was summarized in Table 1” should read “….in the years 2000, 2005, 2011 and 2016 in Ethiopia was summarized in Table 1”, because between indicates that all years from 2000 to 2016.
3. Line 133-136: statements in these lines are vague and do not clearly stated which needs paraphrasing, for instance, what is (61.38, 46.49)% indicate?
4. Does it appropriate to have composite index for these three variables with the consideration of huge proportion difference among the outcome (stunting, wasting and underweight)? For example, there is huge difference on the proportion of stunting and underweight (see table 1). This should well explained in your result section.

Conclusion:

1. Be careful in the use of wording: for instance line 313 " Most prior studies focused on assessing the undernutrition status using stunting ….” Are you Shure that most are not considering the CIAF?
2. The key findings and implications should be summarized instead of repeating the results. Instead the conclusion needs to be made based on the findings and hence it needs correction and modification.
