# Supplementary material for: Disparities in childhood composite index of anthropometric failure prevalence and determinants across Ethiopian administrative zones
Source: PLoS One. 2021 Sep 23;16(9):e0256726. doi: 10.1371/journal.pone.0256726 (PMC8459952; doi:10.1371/journal.pone.0256726)
Supplement: Attachment — Submitted filename: Response to Reviewers.doc [file pone.0256726.s002.doc]

Authors’ Response to Reviewers’ Comments:

*Journal:* Plos One

*Title of paper:* Disparities in childhood Composite Index of Anthropometric Failure prevalence determinants across Ethiopian administrative Zones over time.

Dear editor in chief of PLOS ONE

We thank the editors and reviewers for their constructive comments and suggestions. We have modified the manuscript according to the comments and suggestions made by the editors and reviewers. Our detailed point-by-point response (in blue) to the comments raised by both the editors and the reviewers is given below.

All the questions, suggestions and corrections given by the reviewers are incorporated in the revised manuscript. We believe that our manuscript has been considerably improved and hope that our revised manuscript “**Disparities in childhood Composite Index of Anthropometric Failure prevalence determinants across Ethiopian administrative Zones overtime**” is suitable for publication in the Plos one journal.

Thank you once again for your consideration of our work and for inviting us to submit the revised manuscript. We look forward to hearing from you.

Best regards,

**In response to comments from Reviewer #1:**

General comments

a. Overall, the manuscript is interesting. However, the manuscript might benefit from some revision.

Our response: we have read your comments carefully and made every effort to address them. We generally agree with most of the comments made by the reviewer and we have incorporated corresponding revisions into the revised manuscript. We hope that the manuscript has been improved after this revision.

b. The authors need to show a clear constructing of the composite measure index and compare the composite measure index and the three traditional measures indices (WAZ, HAZ, WHZ). Also, authors should address the question, "what does the difference between the composite indicator measure and the traditional once?

Our response: Commonly, stunting, wasting and under-weight have been conventionally utilized to assess the prevalence of under-nutrition among children. As these indices grossly underestimate this prevalence mainly due to the overlapping of the children into multiple categories of anthropometric failure, there is a need for an appropriate single measure to assess this prevalence and identify the more susceptible individuals. The present study tries to ascertain whether the use of the Composite Index of Anthropometric Failure (CIAF) is more appropriate than the conventional indices for the estimation of under-nutrition among children. Our result (plot added in the revised manuscript) and Table 1 showed that using the conventional indices, the prevalence of under-nutrition for the EDHS 2000 was observed to be 10.70% (wasting), 51.22% (stunting), and 47.10% (under-weight). However, with the use of the CIAF, this prevalence increased to 61.38% and included both single and multiple anthropometric failures. Hence the most important of the CIAF is that it can highlight the severity and seriousness of the actual load of undernutrition in the population.

c. In addition, as they using hierarchical data structure and generalized multilevel mixed model is selected (line 182), the authors need to provide a clear level of the model and the notation of the variables in each level in the method section.

Our response: We have accessed the model and some revision has been made. However, in our analysis, we considered the mixed model instead of the multilevel/hierarchical approach. As it is known that, the multilevel model focuses on designs where all the random factors are nested (like children nested within zones.) which are called levels. But in the case of mixed models, all the random factors would describe as “random factors” that are the zones.

d. DHS **children's data contains** children of women born in the last 5 years, hence, authors how the correlated effects of children from the same women treated?

Our response: Very interesting concern: The DHS data inherent in the sampling weight. To alleviate this issue, we have considered complex survey analysis to avoid biased estimates.

e. On top of these, the title is “Disparities in childhood Composite Index of Anthropometric Failure prevalence determinants across Ethiopian administrative Zones over time” but the effect of time is not investigated/showed, and hence authors should indicated the effect of time on the CIAF prevalence in their results.

Our response: We did the prevalence of CIAF for EDHS 2000, 2005, 2011, and 2016 datasets in Table 1 in the original manuscript and we add the trends in Fig 2 (added in the revised manuscript).

f. Minor: all figures and tables are not clear and needs re-organize with PLOS ONE format. And the references are not following the journal format (e.g.: it is found in font size of 11 and single spacing). Abbreviations with its long form should state at the first time and used only abbreviations after a while, for instance “Composite Index of Anthropometric Failure (CIAF)” is used in whole document with similar fashion whereas “U5C” is in other round way.

Our response: The revised manuscript and figures are revised according to the format of PLOS ONE journal.

**Abstract**

1. Line 6-8: “…..in 2000, 2005, 2011, and 2016” should read “…. (EDHSs) of 2000, 2005, 2011 and 2016, a population-based cross-sectional study of 29,599 under-five year children from 63 Zones.”

Our response: Change has been made according to your comment.

2. Line 14: “The pooled prevalence of CIAF…..” what “Pooled” indicates here? CIAF is already indicates the aggregate value, thus authors should read as “the prevalence of CIAF….”

Our response: corrected accordingly.

**Introduction**

1. Line 39-41: “….different forms of malnutrition” should read “….different forms of undernutrition”. Because, CIAF is computed from wasting, stunting and underweight only and malnutrition is beyond these three measures.

Our response: Thank you for your critical review. Change has been made according to the comment.

2. Line 55-57: “As the administrative Zones are mainly ethnic-based, ….” Is unrelated and unclear statement and should remove from the section.

Our response: Major modification has been made.

**Methods**

1. Line 74-75: “This study was conducted on 29,599 children consisting……” does the study unit children?

Our response: Yes indeed

2. Authors indicate somewhere predictors are considered from child level and women level and the linear complement of the model is given at line 110 wrongly. This is because, child level and women level predictors are not included in the model clearly. Hence it needs clarification!

Our response: We classified the variables as child and household-related covariates for simplicity and interpretation purposes. This is because the main interest of the authors here is focusing on fixed and random effects.

3. Line 119-128: all of these lines are reading about BLUP, the model that authors used in this manuscript is GLMM and the parameter of such model is estimated by either MML or IRWLS or other but not by BLUP. Thus, authors are used wrong methods for parameter estimation and needs critical correction. In general, the model and parameter estimation method is not appropriate for the data and needs refitting and re-estimating the parameters using appropriate model (GMMM) and estimation method (MML or IGWLS or nay other).

Our response: Thank you for your concern. In this paper, we are interested to conduct the generalized linear mixed model instead of multilevel. Hence in the mixed model, we have the fixed and random components. The random component (zones) was estimated by BLUP, but the fixed component (including child, maternal, and household-level covariates) was estimated by the common approach which is BLUE. This is because one of the focuses of the authors in this paper was ranking the random (zones) effects according to their performance in under-nutrition measures. This objective is achieved through the mixed-effects model.

**Results**

1. It would have been useful for the authors to show what percentage or proportion of children were classified as undernourished using each of the measures separately and what new information the composite measure is contributing.

Our response: Thank you for your comment. This was shown in Table 1. Moreover, a trend plot (Fig.2) has been added in the revised manuscript.

2. Line 132: “ …. in the years between 2000 and 2016 in Ethiopia was summarized in Table 1” should read “….in the years 2000, 2005, 2011 and 2016 in Ethiopia was summarized in Table 1”, because between indicates that all years from 2000 to 2016.

Our response: Your comment is incorporated

3. Line 133-136: statements in these lines are vague and do not clearly stated which needs paraphrasing, for instance, what is (61.38, 46.49)% indicate?

Our response: Thank you for your valuable comment. Your comment is well taken and incorporated it in the revised version.

4. Does it appropriate to have composite index for these three variables with the consideration of huge proportion difference among the outcome (stunting, wasting and underweight)? For example, there is huge difference on the proportion of stunting and underweight (see table 1). This should well explained in your result section.

Our response: The conventional under-nutrition measure under-estimate the real burden of the situation. This is mainly due to the overlapping of the children into multiple categories of anthropometric failure, there is a need for an appropriate single measure to assess this prevalence and identify the more susceptible individuals.

**Conclusion**

1. Be careful in the use of wording: for instance line 313 " Most prior studies focused on assessing the undernutrition status using stunting ….” Are you Shure that most are not considering the CIAF?

Our response: Yes as far as our knowledge is concerned, only a few studies had considered it. Even those studies focused on the macro-level (country or regions).

2. The key findings and implications should be summarized instead of repeating the results. Instead the conclusion needs to be made based on the findings and hence it needs correction and modification.

In addition, authors did not include all of the declaration sections and better to include all.

Our response: Thank you. We have revised the paper as per the guidebook of the journal.

**In response to comments from Reviewer #2:**

**Title**

1. The way the tile written is confusing. Is it disparities in prevalence or determinants? The way the finding was written is also not suggestive.

Our response: Thank you for your critical point. The title is corrected in the present version. Moreover, in the present paper we aimed at triple objectives i) determining the real prevalence of under-nutrition (CIAF) ii) identifying the potential risk factors affecting under-nutrition, and iii) looking at the disparities of CIAF among Ethiopian administrative zones.

2. Omit the third affiliation statement on the title page. i.e. “3Department of Statistics, College of Science, Bahir Dar University, Bahir Dar- Ethiopia”.

Our response: Thank you. The correction is made accordingly.

**Abstract**

1. Your first statement on the background and the objective is relatively not conforming. Please make the background more specific.

Our response: correction is made as per the comment.

2. Under result part, I don’t think the word ‘femaleness’ as proper scientific term here. Better to report as ‘being female’ and also some other expressions like “no experience of comorbidity” is also not standard way of expressions.

Our response: The whole document is revised as per the suggestion.

3. On the topic disparity was the main objective. However on the result it was explained as some supplementary finding.

Our response: Thank you. This is a good point and some correction has been made. Our interest was looking at the disparities of CIAF between the zones in Ethiopia. Since zones in the country have the mandate for health-related decisions including nutrition. Therefore, the Fig 6 (maps) shows the disparities of the situation among the Ethiopian administrative zones based on both the crude prevalence and the estimated random effects.

4. Your conclusion should be implication of your finding. For instance, the second statement under the conclusion cannot be you conclusion from this study by any means. Even it cannot be your finding. Rather it might be one justification to do this analysis.

Our response: Thank you. Some corrections and modifications have been done. Moreover, in this paper, our interest was to see the disparities of CIAF among Ethiopian administrative zones. To achieve this aim the generalized mixed model was adopted. In the mixed-model approach, the interest is to find the best linear unbiased predictor, which is used to rank the performance of the random effects. Hence, the conclusion is correct.

5. Revise your keywords. For instance “Adjusted odds ratio” may not be the keyword for this study

Our response: Thank you it is revised as per your suggestion.

**Background**

1. You haven’t explained anything about the determinants of anthropometric failure and why it is needed to study about the issue as it is one of the objectives of this study.

Our response: The use of CIAF over the conventional measures was explained in the introduction part. Moreover, some points are added in the current version.

2. What do other studies say about the CIAF? It could be from study in the other countries.

Our response: Revision is made and included in the revised manuscript (mainly in the introduction and discussion part).

3. You said that administrative zones are ethnic based which is not the case in most parts of the country. In addition the idea of the whole sentence here is not coherent to what is written before it and difficult to understand its implication here with respect to your study

Our response: Thank you for your suggestion. In Ethiopia, Zone's health departments have the mandate to plan, follow up, monitor, and evaluation of health activities at the lower level. So that one of our focuses in this paper was comparing the performance of the administrative zones on childhood under nutritional status.

**Method**

4. Line 63 remove subtitle “data sources”

Our response: Revision has been made as suggested

5. You have did wealth index for urban and rural using different assets for EDHS 2000. How did you combined after you constructed it for use as a variable?

Our response: The study participants of the EDHS dataset are from both rural and urban areas. Since the source of assets varies from rural to urban dwellers, then we take into account this difference to compute the “WI”. In the EDHS, we have considered different items (water, toilet, electricity, radio, television, telephone, refrigerator, motorcycle/scooter, car/truck, cattle, floor, own house, cropland) to measures the WI, some are common for both rural and urban. But some items like “owing cattle” seems more common in rural and some item like a source of energy (electricity) common in urban. We split the dataset into rural and urban areas.

Steps

1. We have encoded all the 11 variables into dichotomous cases (yes (1) and no (0))
2. We checked the frequency of each item and if it is between 5% and 95% it is acceptable to include it in the wealth index calculation for both urban and rural.
3. Principal component analysis (PCA) has been conducted to generate the wealth index scores for urban and rural separately and then merging (mixing) them together.
4. We implement PCA in STATA vs. 14 to construct the wealth index scores.

pca water hv206 hv207 hv208 hv209 hv211 hv212 hv221 sh25ac floor toilet

/*This generates the wealth index scores*/

5. Divide the factor scores into five equal parts (quintiles)

WI= wealth index scored, nq(5). The four cut point values of the quintiles (<20%, 40%, 60% and 80%) with five independent categories (poorest to richest) were considered. Therefore, Create a new variable for the quintiles and recode for those wealth scores values less than or equal to the first cut point, greater than the first and less than or equal to the second cutpoint, greater than the second, and less than or equal to the third cutpoint, greater than the third and less than or equal to the fourth cutpoint, and greater than the fourth cutpoint .

We added how the wealth index is computed with relevant references.

6. How did you score women’s autonomy? Explain it briefly

Our response: More explanation is provided in the revised form of the manuscript. A supplementary file can be provided for details.

7. Was the data weighted? If any, how?

Our response: yes certainly

8. How does CIAF pooled?

Our response: Thank you. The aggregated CIAF was performed using different measures. It is not pooled, it was a typing error.

**Result**

1. On line 141 you wrote table 2 and 3 as they are is reporting a two by two table results but table 2 is only describing one variable each through the EDHS years.

Our response: Major revision has been made on it.

2. Put percentages with one decimal place

Our response: Thank you corrected as per your suggestions

3. The way you put Childhood undernutrition (CIAF) on table 2 is misleading. For example 29,599 looks like total children with CIAF which is not the case.

Our response: Thank you. Now it is corrected and the 95% CI is added

4. What is the justification behind classifying child age in the way you classified on table

Our response: It was the author’s calculation. Now we used the other scenario from a published journal

Risk factors for stunting among childrenunder five years: a cross-sectionalpopulation-based study in Rwanda usingthe 2015 Demographic and Health Survey (classified as: <6, 6-23 and 24-59)

5. Line 149- 152 “Compared to their counterparts, children in the lower age group, in the lower birth order, and without comorbidity associated with a lower prevalence of CIAF. However, the rate was higher among children with a smaller size at birth, and multiple birth types (Table 2)”. To make such findings meaningful you should put at least the confidence interval. Otherwise it is difficult rely on this simple figure to see the difference. For instance is 22.2 and 21.8 really statistically different for the birth order? Even 22.2 might not be different from 17.2 unless we see the confidence interval.

Our response: Thank you for your comments. We corrected the confidence intervals. However, in those tables, we are interested to see the distribution of CIAF for each of the covariates. This is completely descriptive statistics and the statistically significant effect is shown in Fig 3 and 4.

6. Line 155, the expression “Less than half (45.83%) of the women had a lower level of autonomy of decision making in a range of health care situations.” is better if expressed in the other way i.e. “little above half (54.175) of women have average or above autonomy”

Our response: Amended.

7. The way you interpret ORs in the final model findings was not easily understandable and to the standard. Particularly, here you need to extensively edit for language.

Our response: Major revision has been done as your suggestions.

8. On model diagnostics, I am not clear with how you have used VIF to assess the multi-collinearity as this is particularly binary data

Our response: Thank you for your concern. Yes, it was typing error. As you mentioned, multicollinearity works for continuous variables.

**Discussion**

1. Line 253-254 “This difference may be due to ethnic-based variation in geographical, political, and socio-cultural norms…” I don’t think this explanation is evidence based. How many of those included zones were classified based on ethnic based?

Our response: Thank you for your point here. In the Ethiopia most of the zones including (Keffa, Sheka, Bench, Sheko, and Dawro, in Amhara region,,,, Oromoo zone, Awi Waghemera) were classified based on the ethnic groups. However, as per your recommendation, we are talking about the results in terms of second administrative levels (zones).

2. Line 289-290, “ In the Amhara region, it is a common practice to sell more nutritious food items. I think this is not special to Amhara region and may not be strong explanation for higher CIAF

Our response: Thank you. We have modified and tried to justify

**References**
• Check for reference #1 improper use of author initials and #6, #7; using institutions/affiliations just like the way we put the authors name

Our response: managed accordingly

**Figures**
• Some of the texts on your figures are not visible

Our response: Thank you for your critical point. We have managed following PLOS ONE template.

Yours sincerely,

Haile Mekonnen, (on behalf of all the authors)
