# Supplementary material for: Disparities in childhood composite index of anthropometric failure prevalence and determinants across Ethiopian administrative zones
Source: PLoS One. 2021 Sep 23;16(9):e0256726. doi: 10.1371/journal.pone.0256726 (PMC8459952; doi:10.1371/journal.pone.0256726)
Supplement: Attachment — Submitted filename: Response to Reviewers.doc [file pone.0256726.s003.doc]

Authors’ Response to Reviewers’ Comments:

*Journal:* Plos One

***Title of paper:* Disparities in childhood Composite Index of Anthropometric Failure prevalence and determinants across Ethiopian administrative Zones**

Dear editor in chief of PLOS ONE

We thank the editors and reviewers for their constructive comments and suggestions. We have modified the manuscript according to the comments and suggestions made by the editors and reviewers. Our detailed point-by-point response (in blue) to the comments raised by the reviewers is given below.

All the questions, suggestions, and corrections given by the reviewers are incorporated in the revised manuscript. We believe that our manuscript has been considerably improved and hope that our revised manuscript “*Disparities in childhood Composite Index of Anthropometric Failure prevalence and determinants across Ethiopian administrative Zones*” is suitable for publication in Plos one journal.

Thank you once again for your consideration of our work and for inviting us to submit the revised manuscript. We look forward to hearing from you.

Best regards,

**In response to comments from Reviewer #1:**

Reviewer #1: I found that the authors have adequately addressed my comments raised. However, to increase the quality of the manuscript, I recommend a language editing by a native English speaker or by professional.

Our response: Thank you for your recommendation. Professional from English language edited the revised manuscript.

**In response to comments from Reviewer #2:**

General
Language needs to be revised

Thank you for your recommendation. Professional from English language edited the revised manuscript.

**Specific comments**

1. It says “College of Science” for the first author. Is there such college in the University or mistaken?

Our response: Thank you for your concern, yes we have, please visit the college website.

<https://www.bdu.edu.et/cos>
**Abstract**

2. Please re-read the corrections you made for the context …. “Being female of the child….” Also change “no comorbidity” to absence of comorbidity

Our response: Thank you; it has been changed as per your suggestion.

3. Please present the key findings for disparities

Our response: As we stated in the previous revision, one of the interests of the researchers were to see the discrepancies of CIAF in the second administrative levels (zones).To achieve this aim the generalized mixed model was adopted (it is a mixed approach of fixed and random effects). In the mixed-model approach, the interest is to find the best linear unbiased predictor, which is used to rank the performance of the random effects. Hence, the conclusion about the findings is correct with respect to the second administrative zones. The significant effect of zones (random component) is shown in Table 4 and had a statistically significant effect and the distribution of this effect is mapped in Figure 6 compared with the crude prevalence.

4. Your conclusion should be implication of your finding. For instance, the second statement under the conclusion cannot be you conclusion from this study by any means. Even it cannot be your finding. Rather it might be one justification to do this analysis. “Ranking of the performance of the Zones help to target the worst performing, Zones for immediate intervention and the best performing Zones as a role model through implementing the best practice in the national strategy to achieve the SDG2030”. How could we reach to this conclusion the findings you have presented here? Even you removed the better conclusion that we can get from your finding “Disparities of CIAF were observed between and within the Ethiopian administrative Zones over time.” but what does this disparity imply to based on what you have found from the disparity and factors?

Our response: Some revision is considered as per your recommendation and the removed paragraph is added. However, we have considered the zones as random effects (zones) and we reach that random effects are statistically significant. One of the objectives of the mixed model is to look at the effects of both fixed (covariates; independent variables) and random effects, zones in our cases. Particularly, in this paper, one of the objectives is identifying the zones with the best/worst performances of CIAF. This objective is achieved from the random effect components and the result revealed that the given zones have different values of CIAF estimates so that the CIAF disparity conclusion is correct.

**Background**

5. You haven’t explained anything about the determinants of anthropometric failure and why it is needed to study about the issue as it is one of the objective of this study. Which was not addressed on the previous revision.

Our response: Thank you so much for your valuable comments and suggestions. We have revised the manuscript and included all the relevant kinds of literature. This is clearly stated in the introduction part of the manuscript (the whole paragraph).

**Discussion**

6. Your discussion is not as strong as the specific findings of your study. You haven’t well interpreted your findings and the evidence synthesis from your findings was not well established. You simply put so many literatures without strongly discussing about the implication of the findings together. The way you put the explanation for some interpreted findings are merely a simple guess rather than trying to put solid suggestions

Our response: thank you. This is really very important comment. We rewrite the discussion (the whole paragraph is revised) and conclusions based on your suggestions.

**References**
• Your reference also still needs revision. For instance the author name for #1, the way you used et al. please revise based on the requirement for the style you have used

Our response: Thank you. We used Endnote for references and corrected per your suggestions.

Yours sincerely,

Haile Mekonnen, (on behalf of all authors)
